# Isolated Styloid Process Fracture of the Temporal Bone: A Rare Assault-Related Injury

**DOI:** 10.7759/cureus.77876

**Published:** 2025-01-23

**Authors:** Cisel Yazgan, Fatih M Aksakal, Cihat Comert

**Affiliations:** 1 Department of Radiology, University of Health Sciences, Ankara Oncology Training and Research Hospital, Ankara, TUR; 2 Department of Emergency Medicine, University of Health Sciences, Ankara Oncology Training and Research Hospital, Ankara, TUR

**Keywords:** assault-related injuries, craniofacial trauma, eagle’s syndrome, styloid process fractures, temporal bone injuries

## Abstract

Traumatic fractures of the styloid process of the temporal bone, whether related to assault or not, are uncommon injuries and rarely encountered as an isolated entity. The rarity of these fractures often leads to their being overlooked and misdiagnosed. Diagnosing an isolated styloid process fracture requires a high index of clinical suspicion and meticulous evaluation of computed tomography (CT) images. However, it would be challenging without an accompanying maxillofacial fracture. In this report, we present an isolated traumatic fracture of the styloid process in a patient admitted to the emergency department with physical assault and highlight the significance of the multiplanar and three-dimensional (3D)-CT reformatted images.

## Introduction

Traumatic fractures of the styloid process (SP) of the temporal bone are a rare type of injury in patients with maxillofacial trauma and are commonly associated with mandibular fractures [1,2]. The diagnosis of an isolated fracture of SP requires a high level of clinical suspicion, as the clinical presentation can range from no symptoms to facial pain and dysphagia.

The isolated form of SP fractures in patients with maxillofacial trauma is often overlooked, leading to delayed diagnosis and potentially unnecessary treatments. Computed tomography (CT) is a valuable diagnostic tool in detecting fractures of the SP of temporal bone. However, diagnosis can be challenging without an accompanying maxillofacial fracture and requires meticulous evaluation of CT images. In addition to two-dimensional (2D) CT images, the use of three-dimensional (3D) imaging provides detailed visualization of fracture surfaces, enhancing clarity and diagnostic accuracy, which is particularly essential for identifying isolated SP fractures [3].

To the best of our knowledge, only one case of isolated traumatic styloid process fractures without a history of Eagle syndrome has been documented in the English literature [1]. In this report, we present an isolated traumatic fracture of the styloid process in a patient admitted to the emergency department with physical assault and highlight the significance of the 3D CT reformatted images.

## Case presentation

**Figure 1:**
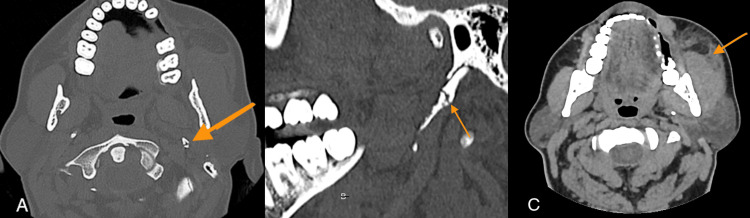
Maxillofacial CT images. Axial (A) and sagittal (B) images show a non-displaced fracture of the left styloid process (yellow arrows). Soft tissue swelling and hematoma in the left masseter muscle are also detected on soft tissue window CT image (C). CT: computed tomography.

**Figure 2:**
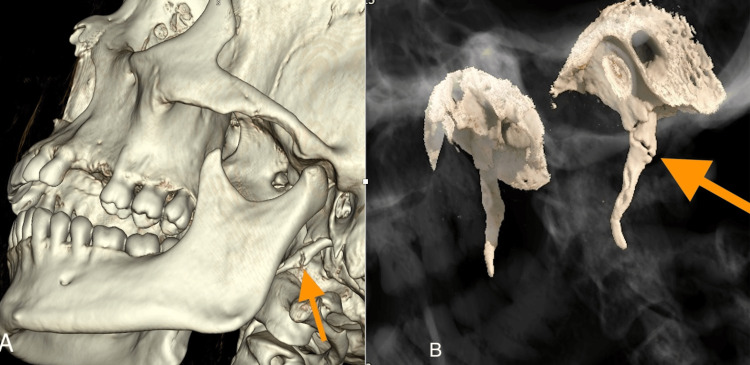
3D reformatted CT images. (A) and (B) Detailed visualization of the fracture surfaces is provided with 3D images (yellow arrows). CT: computed tomography.

A 46-year-old female presented to the emergency department with pain on the left side of her jaw after being punched by her son. There was no loss of consciousness or neurological signs. The patient's Glasgow Coma Scale score was 15 upon admission to the emergency department. There was no history of previous trauma, chronic disease, or medication in the patient's past medical history. Physical examination revealed left facial swelling and tenderness in the retromandibular area. The patient was vitally stable, with no restrictions in mandibular movement. Maxillofacial CT, including multiplanar images (Figures 1A, 1B), identified a non-displaced fracture of the left SP of the temporal bone without accompanying fractures in the mandible or other maxillofacial bones. The 3D images (Figures 2A, 2B) allowed for a more precise evaluation of the fracture surfaces, enabling a better distinction of irregularities. Soft tissue swelling and hematoma in the left masseter and pterygoid muscles were also observed on CT images (Figure 1C).

## Discussion

The SP of the temporal bone is a thin bony prominence extending in an anteroinferior direction from the lower surface of the petrous part of the temporal bone. It is situated between the internal carotid artery (ICA) and the external carotid artery (ECA) and lies posterolateral to the tonsillar fossa. Medially, the SP is adjacent to the internal jugular vein and cranial nerves VII, IX, X, XI, and XII. The tip of the SP is located close to the ECA on its lateral side, while on the medial side, it is near the ICA and the sympathetic chain [4].

There are structural variations of the SP, such as a longer and thinner SP or lateral tip orientation [5]. Elongation, defined as longer than 30 mm in length, is the most common morphological variation of the styloid process, with Guimarães and colleagues reporting a prevalence of 60.2% [6]. The clinical significance of SP elongation lies in its potential to cause Eagle syndrome, a condition characterized by a spectrum of symptoms, including facial or neck pain, dysphagia, and otalgia.

A normally lengthened SP is protected by its location. Nonetheless, elongation makes the SP more vulnerable to both intrinsic and extrinsic trauma [7]. In fact, isolated SP fractures have frequently been reported in patients with an elongated SP [8-11]. In our case, however, the length of the styloid process was normal, and elongation was not considered.

Regarding violent assaults, maxillofacial injuries represent a significant proportion of the overall injuries sustained, with the left side of the face usually being targeted [12,13]. The mandible is the most commonly affected site in these injuries [13]. To our knowledge, no cases of isolated styloid process fractures in victims have been reported.

We propose that in our patient, blunt trauma caused by a punch to the anterior mandible led to posterior displacement of the mandible, which may have indirectly impacted the styloid process of the temporal bone. SP fractures are commonly associated with mandibular fractures [1]. However, in this case, no fractures were detected in the mandible or other facial bones.

Diagnosing isolated fractures of the SP can be challenging, particularly in trauma settings, and requires clinical suspicion and a comprehensive evaluation of imaging. CT is a valuable diagnostic tool in detecting fractures of the styloid process. Segmentation and pseudoarticulation of the styloid process, which can resemble a fracture, cause diagnostic challenges in evaluating CT images. On the other hand, the trauma history of the patient, soft tissue swelling, and fascial muscle hematoma, along with CT findings, raise suspicion of SP fracture.

In addition to conventional 2D CT images, reconstructed images are useful in confirming the diagnosis. 3D reformatted CT images provide detailed anatomical views and complementary information. In our case, 3D reformatted images allowed a more detailed evaluation of the fracture and helped differentiate the fracture from pseudoarticulation. The fracture surfaces, which appeared irregular, were assessed more clearly with the 3D reformatted images. Additionally, we were able to compare the fracture with the styloid process on the opposite side.

The management of non-displaced SP fractures is typically conservative, as these injuries rarely lead to significant complications [14]. However, in cases of delayed or missed diagnosis, patients may be at risk for serious complications such as carotid dissection, facial pain, tinnitus, and dysphagia, increasing the risk of morbidity or mortality. In the presented case, given the non-displaced nature of the fracture and the absence of mandibular or other facial fractures, the patient was managed conservatively with analgesics and supportive measures. Close follow-up was planned to monitor the healing process and ensure the absence of late complications.

## Conclusions

In conclusion, despite its extremely low incidence, an isolated fracture of the SP of the temporal bone should be considered in patients with maxillofacial trauma. In addition to 2D CT images, the use of 3D images is crucial for diagnosing an isolated SP fracture. 3D reformatted images provide a better view for detailed evaluation of the fracture surfaces and help differentiate it from segmentation.
